# FOREWORD

**DOI:** 10.3402/gha.v5i0.20016

**Published:** 2012-11-23

**Authors:** Abdul Rahman Lamin

**Affiliations:** Sector for Social and Human Sciences, UNESCO, Accra, Ghana

It is indeed with great honour and humility that I have accepted the invitation to write the Foreword to this supplement of the Global Health Action, exclusively dedicated to a collection of articles generated from the research project of the INDEPTH Network, Climate and Mortality (CLIMO). I hope that future data analyses will also generate insights on migration as an effect of weather and climate change.

In 2009/10, the Social and Human Sciences Sector of UNESCO, through its office in Ghana, and in the context of its regional activities in Africa, provided modest financial support to INDEPTH Network, which initiated a research activity designed to analyse data on temperature, rainfall, and mortality with a view to a scientific understanding of a potential nexus between and among them, and consequently, use the findings to inform policy making in member states in Africa.

The activity brought together researchers from several HDSS centres in Africa, affiliated with INDEPTH Network, along with counterparts from India and Bangladesh, as well as partners in the North, to use existing data-sets to improve our understanding of climate and mortality. This supplement provides a first step in this direction as it analyses the effect that temperature and rainfall have on the subsequent risk of dying.

The United National Framework Convention on Climate change mandated the Intergovernmental Panel on Climate Change (IPCC) to regularly assess the state of the entire evidence available on the scientific basis of climate change, its impacts on life on the planet including human health and the most effective policy responses. However, this assessment report is only as good as the state of research, which—in the health field—is particularly patchy, as far as low-income countries are concerned.

UNESCO's mission is to contribute to the building of peace, the eradication of poverty, sustainable development and intercultural dialogue through education, science, culture, communication, and information. I am particularly proud that the work to which we modestly contributed to was not only one of knowledge generation but also included a strong component of research capacity strengthening. Young African and Asian scientists were trained in state-of-the art statistical tools and guided in the data analysis process as well as in the skills of writing up the results in scientific papers.

It is my hope that the scientific evidence presented in this supplement will assist policy making in the countries represented in the studies, to tackle the challenges of climate change. I would therefore like to congratulate INDEPTH Network and all the researchers involved in the studies, and hope that future opportunities will continue to make it possible to strengthen our collaboration.

*Abdul Rahman Lamin, PhD* *Programme Specialist* Sector for Social and Human Sciences UNESCO, Accra Ghana

